# The effect of a pre-workout supplement on power performance

**DOI:** 10.1186/1550-2783-12-S1-P24

**Published:** 2015-09-21

**Authors:** Nic Martinez, Bill Campbell, Ryan Colquhoun, Dominic Cochrane, Madison Franek, Daniel Bove, Jeff Dolan, Laura Buchanan, Mallory Johnson, Courtney St Louis, Priscilla Lamadrid, Paul Hinebaugh, Ashkan Attarzadeh, Allison Pingel

**Affiliations:** 1 University of South Florida, Performance & Physique Enhancement Laboratory, Tampa, FL, USA

## Background

Product specific research is necessary for determining the efficacy of dietary supplements comprised of different ingredients. Recently, the pre-workout supplement Assault™ (MusclePharm, Denver, CO, USA) was investigated and demonstrated an ergogenic effect through improved lower body muscular endurance and agility choice reaction. However, continued product specific research is necessary for identifying further potential benefits Assault™ has to offer. The purpose of this study was to determine the impact of Assault™ on anaerobic power measured on a Wingate Cycle Test (WAnT), upper body power via medicine ball put (MBP), and lower body power during a countermovement vertical jump (CMJ).

## Methods

13 male adults (24.3 ± 6.4 years; 179.3 ± 5cm; 83.3 ± 13.1kg) volunteered to participate in this investigation. Each participant was required to visit the laboratory on 4 different occasions separated by approximately 7 days. On the first visit, participants underwent a familiarization session in which they practiced a dynamic warm-up, MBP, CMJ and the WAnT. During their second visit, participants were assessed for height, weight and resting blood pressure, and then performed a dynamic warm-up followed by baseline testing for the performance variables MBP, CMJ, and WAnT. On the third visit, approximately 25 minutes prior to the physical assessments, participants ingested either Assault™ or a placebo and repeated testing of all performance variables following a dynamic warm-up. Approximately 1-week later, participants ingested the alternative supplement and repeated all performance testing in the exact same manner as the prior session. Data were analyzed via a 1-factor [1x3] within-subjects repeated measures analysis of variance (ANOVA) using SPSS version 22.0. Post-hoc tests were analyzed via paired samples t-tests. The criterion for significance was set at p ≤ 0.05.

## Results

The repeated measures ANOVA revealed a significant within-subjects effect for peak (p = 0.003) and mean (p = 0.007) anaerobic power relative to the WAnT. Post-hoc analyses revealed that Assault™ provided a significant increase in anaerobic peak power (782 ± 191W) and mean power (569 ± 133W) in comparison to the placebo (p = 0.003; 722 ± 208W), (p = 0.006; 535 ± 149W) and baseline trials (p = 0.011; 723 ± 205W), (p = 0.020; 538 ± 148W), respectively. Table 1 demonstrates the raw data (mean ± SD) in anaerobic peak, mean, and minimum power for each treatment group. No main effects were observed for the MBP (p = 0.314) or the CMJ (p = 0.150).

**Table 1:** Anaerobic Power and Power Drop in Watts (mean ± SD) for each group.

	Peak Power Watts	Mean Power Watts	Minimum Power Watts	Power Drop Watts
Assault™	782 ± 191^#*^	569 ± 133^#*^	356 ± 83	426 ± 145^#^

Placebo	722 ± 208	535 ± 149	336 ± 87	387 ± 135

Baseline	723 ± 205	538 ± 148	331 ± 90	391 ± 148

^# ^- Post-hoc statistical difference compared to baseline values (p ≤ 0.05)

^* ^- Post-hoc statistical difference compared to placebo values (p ≤ 0.05)

## Conclusions

Ingestion of the a pre-workout supplement (Assault™) lead to significant improvements in anaerobic peak and mean power values as compared to the placebo treatment and baseline measures. These elevations came with no adverse effects relative to blood pressure values. Taken prior to exercise, Assault™ supplementation may improve anaerobic power values, thus leading to enhanced performance.

